# Characterization, and Functional Analysis of Hsp70 and Hsp90 Gene Families in *Glyphodes pyloalis* Walker (Lepidoptera: Pyralidae)

**DOI:** 10.3389/fphys.2021.753914

**Published:** 2021-10-21

**Authors:** Jian-hao Ding, Lu-xin Zheng, Jie Chu, Xin-hao Liang, Jun Wang, Xiao-wen Gao, Fu-an Wu, Sheng Sheng

**Affiliations:** ^1^Jiangsu Key Laboratory of Sericultural Biology and Biotechnology, School of Biotechnology, Jiangsu University of Science and Technology, Zhenjiang, China; ^2^Key Laboratory of Silkworm and Mulberry Genetic Improvement, Ministry of Agriculture and Rural Affairs, Sericultural Research Institute, Chinese Academy of Agricultural Sciences, Zhenjiang, China; ^3^Zhenjiang Runyu Biological Science and Technology Development Co., LTD., Zhenjiang, China

**Keywords:** *Glyphodes pyloalis*, heat shock protein, environmental stress, RNA interference, parasitisation

## Abstract

*Glyphodes pyloalis* Walker (Lepidoptera: Pyralidae) is a destructive mulberry pest, causing great damage to mulberry in China. Heat shock proteins (Hsps) are involved in various signal pathways and regulate lots of physiological processes in insects. The function of Hsps in *G. pyloalis*, however, has still received less attention. Here, we identified five Hsp genes from *G. pyloalis* transcriptome dataset including two Hsp70 family genes (*GpHsp71.3* and *GpHsp74.9*) and three Hsp90 family genes (*GpHsp82.4*, *GpHsp89*, and *GpHsp93.4*). Quantitative Real-time PCR validation revealed that all Hsps of *G. pyloalis* have significant expression in pupal and diapause stage, at which the larvae arrest the development. Expressions of *GpHsp71.3* and *GpHsp82.4* were increased significantly after thermal treatment at 40°C, and this upregulation depended on heat treatment duration. Furthermore, silencing *GpHsp82.4* by RNA interference led to a significant increase in mortality of *G. pyloalis* larvae under the heat stress compared to the control group. After starvation stress, the expression levels of *GpHsp82.4* and *GpHsp93.4* were significantly increased. At last, after being parasitized by the parasitoid wasp *Aulacocentrum confusum*, Hsp70 and Hsp90 genes of *G. pyloalis* were decreased significantly in the early stage of parasitization and this moderation was affected by time post-parasitization. This study highlights the function of *G. pyloalis* Hsps in response to environmental stress and provides a perspective for the control of this pest.

## Introduction

Heat shock protein (Hsp) is a highly conservative protein induced by extreme temperature, heavy metals, hunger, and other stresses in organisms, which can help the protein fold correctly, degrade the denatured protein, and exercise the function of molecular chaperone in the biological resistance to adverse environments (Tammariello et al., 1999). In insects, four major families of Hsps, along with several co-chaperones, are recognised, including the small heat shock proteins (sHsps), Hsp60, Hsp70, and Hsp90 (Kim et al., 1998). Hsp70 (72–80 kDa) and Hsp90 (81–99 kDa) are the two most widely studied Hsp families in insects (Feng et al., 2010). Due to the development of genomics, transcriptomics, and other sequencing technologies, an increasing number of Hsps have been reported in insects. For examples, Five *CsHsp70* and *CsHsp90* genes in *Chilo suppressalis* were identified (Lu et al., 2014), as well as two *PxHsp90* and eight *PxHsp70* genes in the *Plutella xylostella* genome (Xia et al., 2013). Hsp70 and Hsp90 function as molecular chaperones in response to a variety of stress factors, promote proper protein folding, and prevent the aggregation of denatured protein (Feder and Hofmann, 1999). Hsps have been found to be involved in many developmental processes, including insect diapause, metamorphosis, embryogenesis, apoptosis, and autophagy (Chu et al., 2020). Hsp synthesis in insects is differently regulated not only at series of developmental stages but also during the diapause process (Sasibhushan et al., 2013). The expression levels of Hsp70 were greatly increased in diapausing pupae of the blueberry maggot, *Rhagoletis mendax* (Teixeira and Polavarapu, 2005). In *Omphisa fuscidentalis*, *OfHsp90* expression increased transiently during the termination of diapause (Tungjitwitayakul et al., 2008).

An important determinant of insect abundance and distribution is temperature, with extremes of heat and cold eliciting adaptive induction of Hsp gene expression required for survival (King and MacRae, 2015). When insects are threatened by extreme temperatures and other stresses, the synthesis of most proteins decreases but the synthesis of highly sensitive proteins increases, binding abnormal proteins and helping them to refold (Kim et al., 1998). Temperature is one of the important environmental factors. The heat resistance of insects is closely related to Hsp synthesis. Hsps in organisms can help to obtain the adaptability of high-temperature environments, improving thermal tolerance (Wang et al., 2018). Studies demonstrate that the regulation of Hsp genes varies with the intensity of thermal stress and the insect’s physiological state. *P. xylostella* showed a significant increase in the expression of *PxHsp90* after heat stress (37°C) (Sonoda et al., 2006). While in *Delia antiqua*, low amounts of Hsp70 mRNA are exhibited, which builds up on exposure to heat and cold, followed by a gradual decline if cold persists (Chen et al., 2006). High and low temperatures boost Hsp90 mRNA in diapausing pupae of *D. antiqua* (Chen et al., 2005). In *Ectomyelois ceratoniae*, the mortality rates significantly increased in *dsHsp90* injection larvae after thermal treatment, suggesting that the *HSP90* plays an important role in conferring thermotolerance (Farahani et al., 2020). According to Rinehart et al. (2007), the deletion of Hsp70 mRNA reduces heat hardiness and cold hardiness in *Sarcophaga crassipalpis*, showing the role of Hsp in heat stress tolerance. In addition, Silence of *PaHsp70* mRNA hinders heat shock recovery and repair of chilling injuries in adult *Pyrrhocoris apterus* (Koštál and Tollarová-Borovanská, 2009). Intermittent food shortages are often encountered by insects. During winter or under starvation stress, insects enter diapause stage as eggs, mature larvae, pupae, or adults (Zhang et al., 2019). In face of starvation, insect responses include changes in behavior and/or maintenance of low metabolic rates through physiological adaptation or regulation. Hsp are involved in these processes that maintain lower levels of physiological metabolic activity (King and MacRae, 2015). Silencing *Hsp70* resulted in lower survivorship after food deprivation in *Rhodnius prolixus* (Paim et al., 2016). Starvation of 24 h can induce the expression of genes encoding *Hsp90* in the endoparasitoid wasp, *Pteromalus puparum* (Wang et al., 2012).

Parasitoid wasps can be the biotic stressor for their host insects and can manipulate the development of the host in order to create a safe environment for their offspring (Rinehart et al., 2002). It has been suggested that there is a functional connection between host response and heat shock regulatory systems (Tutar and Tutar, 2010). For example, upregulation of Hsps in the flesh fly, *S. crassipalpis* in response to venom injected by the ectoparasitoid wasp *Nasonia vitripennis* (Rinehart et al., 2002). Hsp75 and Hsp90 of *Pieris rapae* were downregulated at 12 h post-parasitization and the minimum level was reached at 48 h during the time interval observed in response to parasitization by *P. puparum* (Zhu et al., 2013). Despite this, the function of Hsp in the response of host insects to parasitization stress still needs more exploration and verification.

The mulberry pyralid caterpillar, *Glyphodes pyloalis*, is a major pest of mulberry, causing serious losses to the production of mulberry leaves in China and threatens the development of the sericultural industry every year. *G. pyloalis* occurred four to seven generations in the mulberry-producing areas of eastern and southern China every year. It mainly outbreaks in summer or early autumn when the temperature gets as high as 42°C and it has strong adaptability to this thermal stress (Chu et al., 2020). The high temperature can alter the expression of many genes in *G. pyloalis* (Liu et al., 2017). *G. pyloalis* larvae can enter diapause stage when lacking of food in environment (Chen et al., 2015). Previous studies showed that sHsp are involved in response to high temperature stress in *G. pyloalis* (Chu et al., 2020), however, whether other Hsp families are involved in the response to these genes at high temperatures remains unknown. Meanwhile, it is urgent to elucidate the roles of Hsps when *G. pyloalis* encounter thermal stress as well as other environmental stresses, such as food deprivation and parasitization by their natural enemies.

In this study, we characterized Hsp70 and Hsp90 genes of *G. pyloalis* from transcriptome database and described their structural properties. The expressions of *GpHsps* in response to thermal and starvation stress were analyzed by RT-qPCR The impact of *Aulacocentrum confusum* parasitization on *GpHsps* expression was also examined. In addition, RNAi was used to study the functions of temperature-induced related Hsp genes. Thus, these results will further illustrate the molecular mechanism of insect *Hsps* in response to abiotic and biotic environmental stress. This study may also provide new targets and approaches for the control of *G. pyloalis*.

## Materials and Methods

### Insect Rearing

*Glyphodes pyloalis* larvae were collected from the leaves of a mulberry field at Sericulture Research Institute of the Chinese Academy of Agricultural Sciences in Zhen Jiang, China (32.20°N, 119.45°E). Collected larvae of *G. pyloalis* were reared in laboratory at 26 ± 1°C and 50 ± 5% relative humidity with a 16:8 h light/dark photoperiod and supplied with fresh mulberry leaves in boxes (15 cm × 10 cm × 8 cm) till pupation. After emergence, the male and female adults of *G. pyloalis* were transferred into a transparent glass jar (30 cm × 20 cm × 10 cm) for mating and spawning. *A. confusum* was obtained from rearing parasitized *G. pyloalis* larvae in the mulberry field. *A. confusum* were maintained using the larvae of *G. pyloalis* as the hosts. Adult wasps were kept in glass tube and fed with a 10% (w/v) honey solution.

### Identification and Bioinformatic Analysis of Hsp Genes in *G. pyloalis*

The Hsp70 and Hsp90 genes were identified from the previously constructed transcriptome dataset (ACC.SRR9959773, Sheng et al., 2021). ORFfinder^1^ was used to predict the open reading frames (ORFs) of *GpHsps* and CLUSTALX analysis was conducted to obtain aligned amino acid sequences. ExPASy^2^ was used to predict the theoretical isoelectric point (pI) and molecular weight of each enzyme. Conserved motifs and domains were predicted using PFAM^3^ and SMART.^4^ The phylogenetic tree of Hsp70 and Hsp90 in *G. pyloalis* Walker and other insects were generated using neighbor-joining for 1000 times bootstrap replicates by MEGA 7 software.

### Sample Preparation for Real-Time Quantitative PCR

The sample design for the initial experiment has been described previously. Two-day old 1st instar, 3rd instar, and 5th instar larvae, pupae, and male and female adults were collected and stored at −80°C until RNA extraction. The fifth instar larvae were collected and reared at 16°C for 7–10 days. Once the larvae molted into the pre-pupal stage to enter into the diapause, they were collected and kept for half a month without pupation to obtain diapause larvae. In the thermal treatment, 2-day old 5th instar larvae were selected for heat and cold shock, and three temperature levels (40, 25, and 0°C) were imposed on the larvae. To evaluate the effect of exposure time on gene expression, two levels of exposure duration (1 and 4 h) were examined for their short- and long-term effects. In the starvation treatment, 2-day old 4th instar *G. pyloalis* larvae were deprived for food for various times (0, 2, 4, and 6 days) to obtain the cohorts of starvation larvae. Control groups were provided with mulberry leaves. After starvation treatment, the surviving larvae were frozen and stored at −80°C as described above. Each treatment included at least three surviving larvae. The parasitization experiment was conducted following the methods described by Zheng et al. (2017). The parasitic conditions were as follows: twenty 4th larvae of *G. pyloalis* were placed into a transparent circular plastic box (90 × 90 × 100 cm) with a single mulberry leaf at the bottom, under which the mulberry leaves were moisturized with agar layer. One hour later, a mated female parasitoid was introduced into the box. After parasitism for 6 h, the female wasps were removed, and the larvae were collected separately and fed in plastic cups (5 × 5 × 2.5 cm). After the parasitoid cocoons formed, the parasitoids were taken out and placed in glass tubes separately till the adults emerged. These larvae at 0, 2nd, 4th, or 6th day after parasitization were collected and stored at −80°C until RNA extraction.

### Extraction of Total RNA From *G. pyloalis* and Synthesis of cDNA

Total RNA samples were extracted using the Trizol reagent (Invitrogen, Carlsbad, CA, United States) following the manufacture’s protocol, and the residual DNA was removed by RNase-Free DNase I (Promega, Madison, WI, United States). The integrity of total RNA was detected by 1% agarose gel electrophoresis, and the concentration and purity of total RNA from *G. pyloalis* were determined by Nanodrop 2000 spectrometer (Thermo Scientific, Waltham, MA, United States). All RNA samples were reverse transcribed using the PrimeScript RT reagent kit with gDNA Eraser (Takara Biotechnology Co. Ltd., Dalian, China). The qualified cDNA was stored at −20°C for later use.

### Real-Time Quantitative PCR

Real-Time Quantitative PCR (RT-qPCR) was performed using QuantStudio^TM^ Real-Time PCR system (Applied Biosystems, Foster, CA, United States). Each reaction was conducted in a 20 μL volume that consisted of the following: 10 μL SYBR Premix Ex Tap II (Takara Biotechnology Co. Ltd., Dalian, China), 0.4 μL each of the forward and reverse primer, 0.4 μL ROX Reference Dye (50×), 1.5 μL cDNA template, and 7.3 μL ddH2O. The beta-actin gene (MK450335.1) was used as the reference gene to normalize the expression of target genes and correct variation. Following RT-qPCR, the reaction process was as follows: 5 min at 95°C, followed by 45 cycles of 15 s at 95°C, 60°C for 31 s, and dissociation. A fluorescence melting curve from 55 to 95°C was used to ensure a single gene-specific peak and the absence of primer-dimer peaks. Each PCR reaction was replicated three times. The primers used in RT-qPCR analysis were designed by Primer-BLAST^5^ and are listed in Supplementary Table 1. The relative expression levels of *GpHsps* among the different samples were measured using 2^–ΔΔCt^ method.

### dsRNA Synthesis and Injection and Mortality Bioassay

Primers (Supplementary Table 2) of dsRNA of *GpHsp71.3* and *GpHsp82.4* were designed with BLOCK-i^TM^ RNAi Designer.^6^ Green fluorescent protein (GFP) was selected as a negative treatment. According to the manufacturer’s instructions, the dsRNA was synthesized using an *in vitro* Transcription T7 Kit (for siRNA Synthesis) (Takara Biotechnology Co. Ltd., Dalian, China). The integrity of the dsRNA was determined by 1% agarose gel electrophoresis. The quality and concentration of purified dsRNA were measured using NanoDrop 2000 spectrophotometer. To determine the RNAi efficiency, *dsGpHsp71.3* and *dsGpHsp82.4* were, respectively, dissolved to a final concentration of 1.0 μg/μL and 5th instar 2-day old larvae were selected for treatment and injection of 0.5, 1, 1.5, and 2 μL of dsGFP/dsRNA, respectively, using a Nanoject II microinjector (Drummond Scientific, Broomall, PA, United States). Triple biological replicates were done for each group. After 24 h of injection, both dsGFP and dsHsp larvae were collected and stored at −80°C as described above for RT-qPCR. RT-qPCR was used to check the efficiency of RNAi. Mortality rates of larvae of *G. Pyloalis* after RNAi of *GFP* or *GpHsp* were determined under heat shock treatment. After 24 h of injection, thirty 2-day old 5th instar larvae of *G. pyloalis* were exposed to 40°C for 1 h for larvae of *dsGFP/dsGpHsp82.4* and 4 h for larvae of *dsGFP/dsGpHsp71.3* to determine the effect of heat temperatures on survival, respectively. Mortality of larvae after heat shock treatment was recorded for 48 h separately.

### Statistical Analysis

A one-way analysis of variance (ANOVA) (Systat, Inc., Evanston, IL, United States) with Tukey’s *post hoc* test (*p* < 0.05) was used to check for significant differences in the expression levels of each target gene among the treatments. A two-way ANOVA is used to estimate significant differences in the expression levels affected by parasitization and durations post-parasitization. Chi-square teat was used to analyses mortality in bioassay. The data analysis was carried out in R 4.0.4 software (R Core Team, 2013).

## Results

### Sequences of Hsp70 and Hsp90 in *G. pyloalis*

**FIGURE 1:**
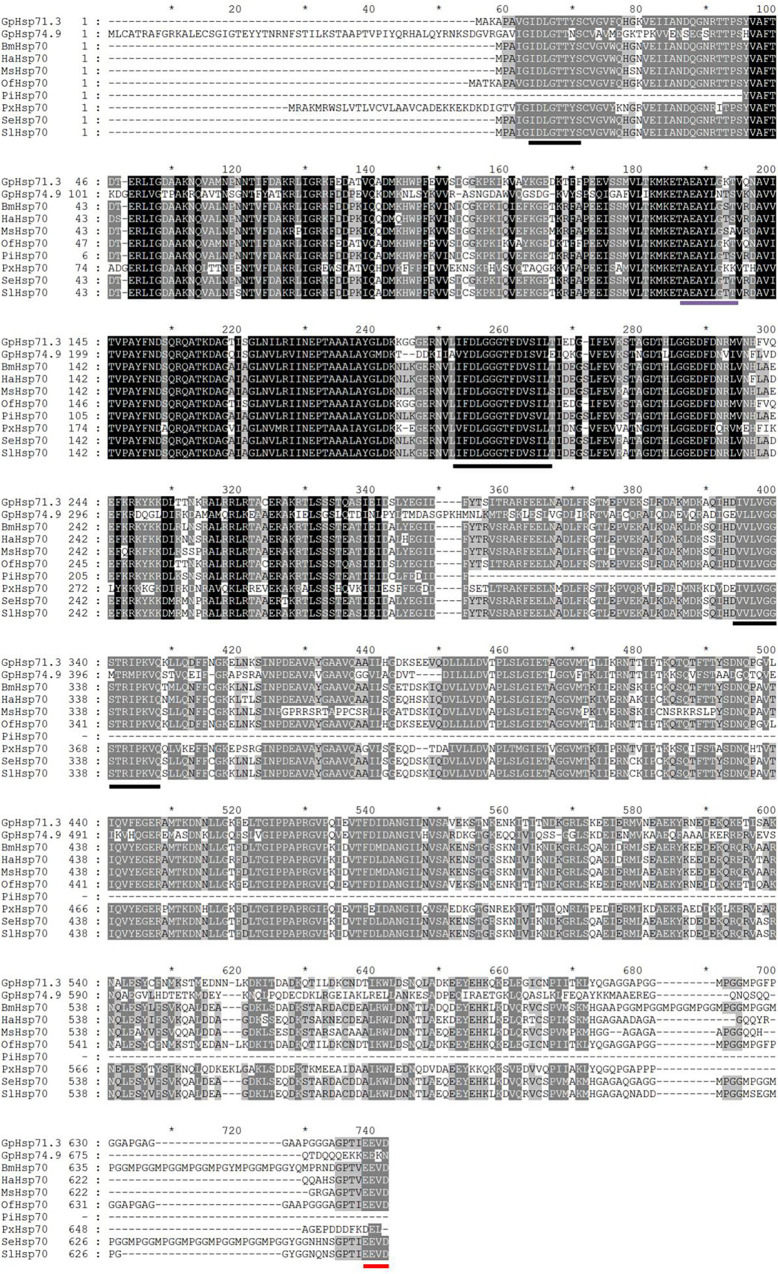
Alignment of the deduced amino acid sequences of Hsp70 genes in *G. pyloalis* and other Lepidoptera insect. The conserved domains of Hsp70 gene family is underlined in Black solid line. Red solid line indicates a conserved amino acid sequence of EEVD motif at C-terminus and purple solid line indicates a ATP/GTP binding site motif. *Bombyx mori* (Bm), *Helicoverpa armigera* (Ha), *Manduca sexta* (Ms), *Ostrinia furnacalis* (Of), *Plodia interpunctella* (Pi), *Plutella xylostella* (Px), *Spodoptera exigua* (Se), *Spodoptera litura* (Sl).

**FIGURE 2:**
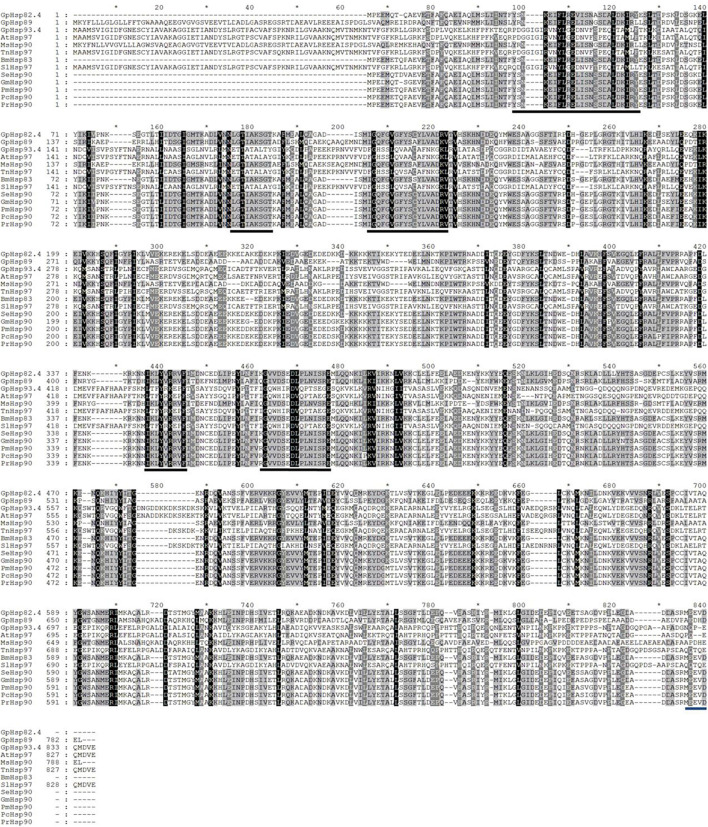
Alignment of the deduced amino acid sequences of Hsp90 genes in *G. pyloalis* and other Lepidoptera insect. The conserved domains of Hsp90 gene family is underlined in Black solid line. Blue solid line indicates a conserved amino sequence of MEEVD motif at C-terminus. *Amyelois transitella* (At), *Mythimna separata* (Ms), *Trichoplusia ni* (Tn), *Bombyx mori* (Bm), *Spodoptera litura* (Sl), *Spodoptera exigua* (Se), *Grapholita molesta* (Gm), *Pieris melete* (Pm), *Pieris canidia* (Pc), *Pieris rapae* (Pr).

Two Hsp70 genes (*GpHsp71.3* and *GpHsp74.9*) and three Hsp90 genes (*GpHsp82.4*, *GpHsp89*, and *GpHsp93.4*) were identified, and the GenBank accession numbers were MK225533–MK225537. All GpHsp genes contained full-length Open Reading Frame, encoding 652–837 amino acid residues with predicted isoelectric point (pI) of 4.97–5.96 and molecular weight ranges from 71.3 to 93.4 kDa (Table 1). Alignment of the deduced amino acid sequences of ten Hsp70 in *G. pyloalis* and other lepidopterous insects showed that *GpHsp70s* have great similarities with the Hsp70 family of other lepidopteran insects (Figure 1). Both *GpHsp70* have three signature motifs of the Hsp70 family: IDLGTTYS (amino acids 64–71), IFDLGGGTFDVSLL (amino acids 253–266), and IVLVGGSTRIPKVQQ (amino acids 395–409). Besides, they have a unique ATP/GTP binding site (AEAYLGKN) at amino acids 187–194 and a conservative amino acid sequence of EEVD motif at C-terminus. Multiple sequence alignments of Hsp90 showed a high similarity between species as shown in Figure 2. *GpHsp90* has five signature sequences of the Hsp90 protein family: YSNKEIFLRELISNSSDALDKIR (residues 98–120), LGTIAKSGT (residues 167–175 residues), IGQFGVGFYSAYLVAD (residues 197–212), IKLYVRRVFI (residues 430–439), and GVVDSEDLPLNISRE (residues 456–470) and possess a conserved sequence MEEVD motif (residues 852–856) at the C-terminus. *GpHsps* displayed high amino acid identity with the Hsps of *Mythimna separata* (88%), *Ostrinia furnacalis* (88%), *M. separata* (87%), *Spodoptera exigua* (81%), and *Amyelois transitella* (79%) (Table 1).

**TABLE 1:** Sequence characteristics of Hsp70 and Hsp90 mRNA in *Glyphodes pyloalis*.

**Gene name**	**GenBank accession number**	**ORF length (aa)**	**Molecular weight (kDa)**	**Isoelectric point**	**BLASTX best hit**				
					**Species**	**Gene name**	**Accession number**	***E*-value**	**Identify (%)**
*GpHsp71.3*	MK225533	652	71297.54	5.32	*Mythimna separata*	Heat Shock Protein 70	EU306518.1	0	88
*GpHsp74.9*	MK225534	687	74883.74	5.96	*Spodoptera exigua*	Heat Shock Protein 70	FJ609645.1	0	81
*GpHsp82.4*	MK225535	716	82379.32	4.99	*Ostrinia furnacalis*	Heat Shock Protein 90	GU230734.1	0	88
*GpHsp89*	MK225536	783	88998.12	4.97	*Mythimna separata*	Heat Shock Protein 90	KX977591.1	0	87
*GpHsp93.4*	MK225537	837	93392.05	5.51	*Amyelois transitella*	Heat Shock Protein 90	XM_013330196.1	0	79

### Phylogenetic Analysis

**FIGURE 3:**
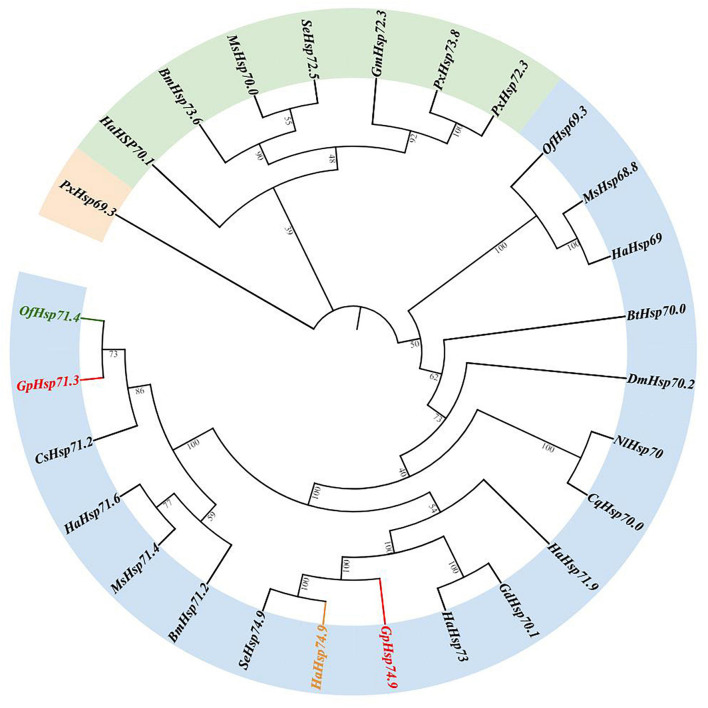
The neighbor-joining tree of *GpHsp70s* deduced from the coding sequence (CDS) between *G. pyloalis* and other insect species. *GpHsp71.3* and *GpHsp74.9* in red, *OfHsp71.4* in green, and *HaHsp74.9* in orange. *Bombyx mori* (Bm), *Helicoverpa armigera* (Ha), *Manduca sexta* (Ms), *Ostrinia furnacalis* (Of), *Plutella xylostella* (Px), *Spodoptera exigua* (Se), *Bemisia tabaci* (Bt), *Nilaparvata lugens* (Nl), *Harmonia axyridis* (Ha), *Galeruca daurica* (Gd), *Drosophila melanogaster* (Dm), *Culex quinquefasciatus* (Cq), *Chilo suppressalis* (Cs), *Grapholita molesta* (Gm).

**FIGURE 4:**
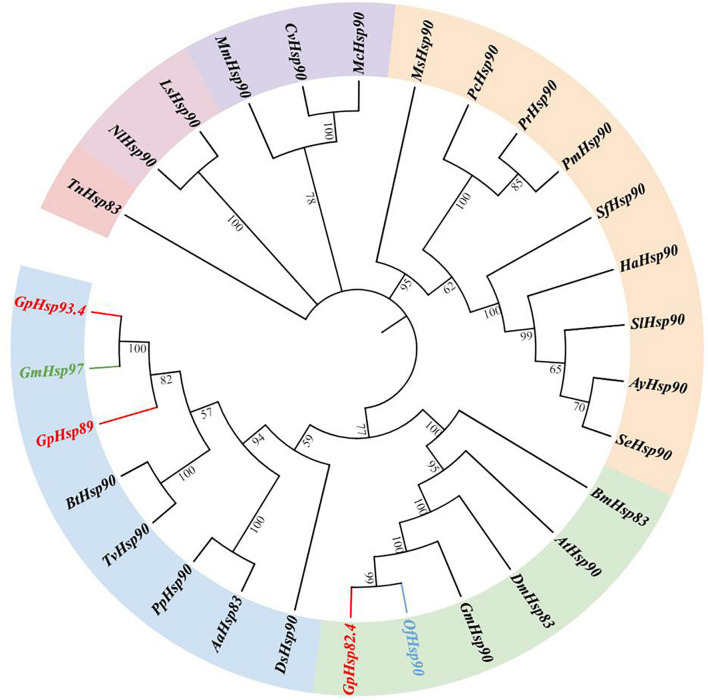
The neighbor-joining tree of *GpHsp90s* deduced from the coding sequence (CDS) between *G. pyloalis* and other insect species. *GpHsp82.4, GpHsp89, and GpHsp93.4* in red. *OfHsp90* in blue and *GmHsp97* in green. *Trichoplusia ni* (Tn), *Amyelois transitella* (At), *Helicoverpa armigera* (Ha), *Ostrinia furnacalis* (Of), *Microplitis mediator* (Mm), *Spodoptera litura* (Sl), *Plutella xylostella* (Px), *Spodoptera exigua* (Se), *Antheraea yamamai* (Ay), *Pieris canidia* (Pc), *Spodoptera frugiperda* (Sf), *Pieris rapae* (Pr), *Dendrolimus superans* (Ds), *Manduca sexta* (Ms), *Bombyx mori* (Bm), *Grapholita molesta* (Gm), *Pieris melete* (Pm), *Aedes aegypti* (Aa), *Laodelphax striatellus* (Ls), *Bemisia tabaci* (Bt), *Trialeurodes vaporariorum* (Tv), *Cotesia vestalis* (Cv), *Pteromalus puparum* (Pp), *Nilaparvata lugens* (Nl), *Macrocentrus cingulum* (Mc), *Harmonia axyridis* (Ha), *Drosophila melanogaster* (Dm).

Results of the phylogenetic tree showed that *GpHsp71.3* and *GpHsp74.9* were clustered into separate clusters and were adjacent with *OfHsp71.4* and *HaHsp74.9*, respectively (Figure 3). Among three *GpHsp90* (*GpHsp82.4, GpHsp89, and GpHsp93.4*), *GpHsp82.4, and OfHsp90* were grouped into one cluster, while *G. pylHsp89* and *GpHsp93.4* was adjacent with *GmHsp97* (Figure 4).

### Expressions of *GpHsps* in Different Developmental Stages

**FIGURE 5:**
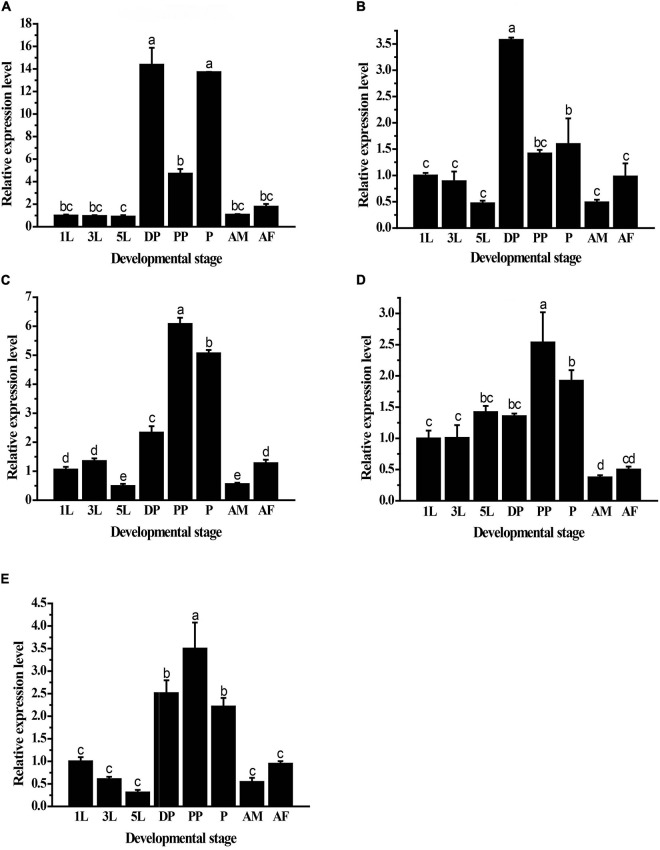
Relative mRNA expression levels of five *GpHsps* in different developmental stages. **(A)** *GpHsp71.3;* **(B)** *GpHsp74.9;* **(C)** *GpHsp82.4;* **(D)** *GpHsp89;* **(E)** *GpHsp93.4*. 1st instar larvae (1L), 3rd instar larvae (3L), 5th instar larvae (5L), diapause larvae (DP), prepupae (PP), pupa (P), male adults (AM), and female adults (AF) of *G. pyloalis*. The relative expression levels of the *GpHsps* were determined by comparison with the first instar larvae. Data are shown as the mean ± SE of three biological replicates. Differences among triple repeats were analyzed using one-way analysis of variance (Systat, Inc., Evanston, IL) with Tukey’s *post hoc* test. The different letters above the bars indicate significant differences (*p* < 0.05).

The results showed significant differences in the expression levels of *GpHsps* at different developmental stages. *GpHsp71.3* was highly expressed in diapause larvae and pupae, and the expression of *GpHsp74.9* was the highest at pupal stage. *GpHsp82.4* and *GpHsp89* were highly transcribed in pre-pupae and pupae. The expression levels of *GpHsp93.4* were exhibited more significantly in pre-pupae, pupae, and diapause stages than the other developmental stages (Figure 5).

### Expression of *GpHsps* Under Temperature Stress

**FIGURE 6:**
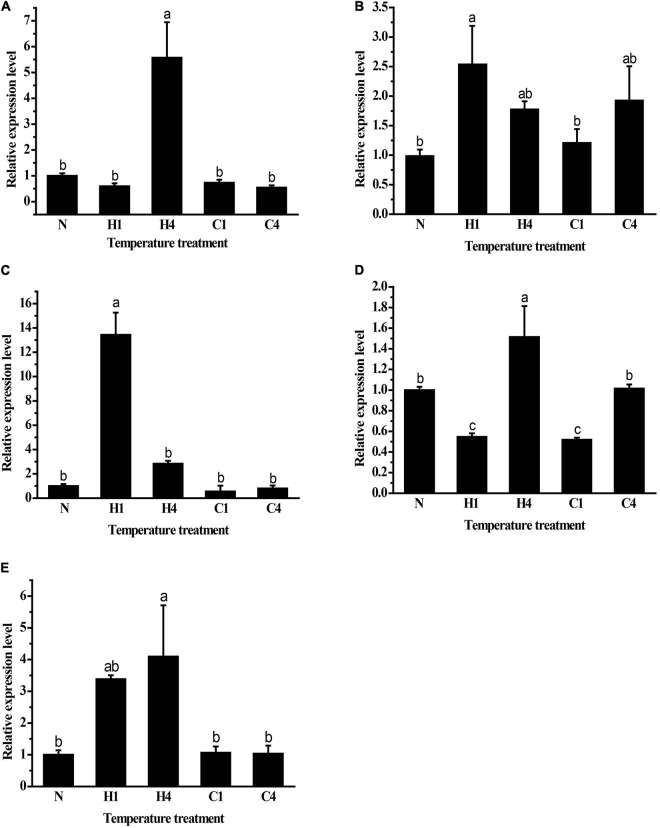
Relative mRNA expression levels of *GpHsps* at extreme cold and heat temperature stress. **(A)** *GpHsp71.3;* **(B)** *GpHsp74.9;* **(C)** *GpHsp82.4;* **(D)** *GpHsp89;* **(E)** *GpHsp93.4*.N: normal temperature control (25°C); H1: heat shock (40°C) for 1 h, H4: heat shock (40°C) for 4 h, C1: cold shock (0°C) for 1 h, C4: cold shock (0°C) for 4 h. Data are shown as the mean ± SE of three biological replicates. Differences among triple repeats were analyzed using one-way analysis of variance (Systat, Inc., Evanston, IL) with Tukey’s *post hoc* test. Different lowercase letters indicate significant differences (*p* < 0.05).

The expression patterns of *GpHsp70s* and *GpHsp90s* after exposure to cold (0°C) and heat (40°C) stress were analyzed. The expression of *GpHsp74.9*, *GpHsp82.4*, and *G.pHsp93.4* were upregulated significantly after 1 h of high temperature stress at 40°C. *GpHsp71.3* and *GpHsp89* expression increased after 4 h at 40°C. The expressions of Hsp genes did not show significance under cold stress treatment, except for *GpHsp74.9*, which were upregulated compared to the control group after 4 h of treatment. These results indicate that *GpHsps* respond to temperature stress and are especially responsive to extreme temperatures but more susceptible to heat temperature induction (Figure 6).

### Expressions of *GpHsps* in Response to Starvation Stress

**FIGURE 7:**
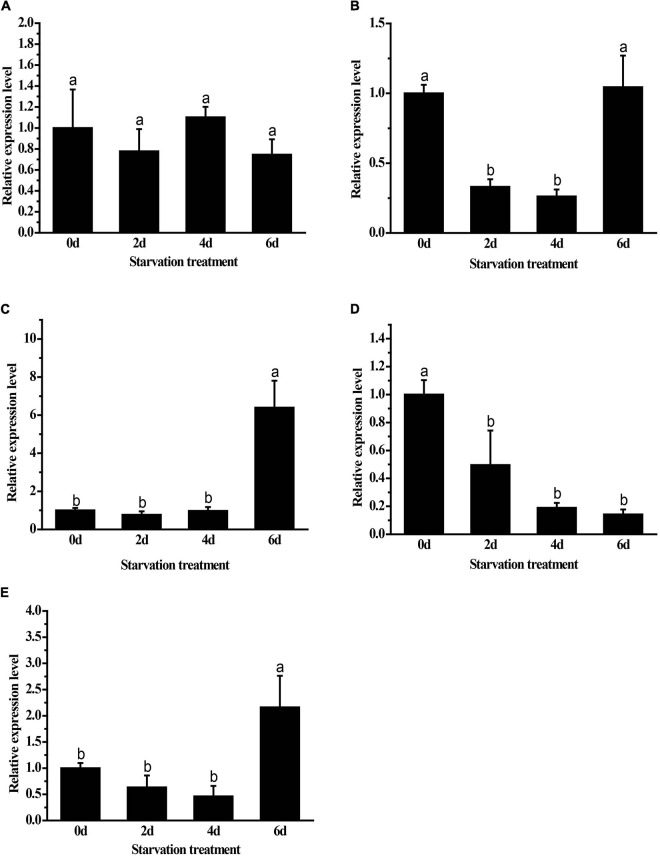
Expression levels of *GpHsps* after starvation stress. **(A)** *GpHsp71.3*; **(B)** *GpHsp74.9*; **(C)** *GpHsp82.4*; **(D)** *GpHsp89*; **(E)** *GpHsp93.4*. Data are shown as the mean ± SE of three biological replicates. Differences among triple repeats were analyzed using one-way analysis of variance (Systat, Inc., Evanston, IL) with Tukey’s *post hoc* test. Different lowercase letters indicate significant differences (*p* < 0.05).

Both *GpHsp82.4* and *GpHsp93.4* showed significantly upregulated expression after 6 days of food deprivation, and the expression levels of *GpHsp74.9* were downregulated at second day and fourth day after food deprivation while *GpHsp71.3* is unaffected by starvation treatment. In contrast, the expression of *GpHsp89* showed an opposite pattern with the increase in time (Figure 7).

### *GpHsps* Expression During Parasitism by *A. confusum*

**FIGURE 8:**
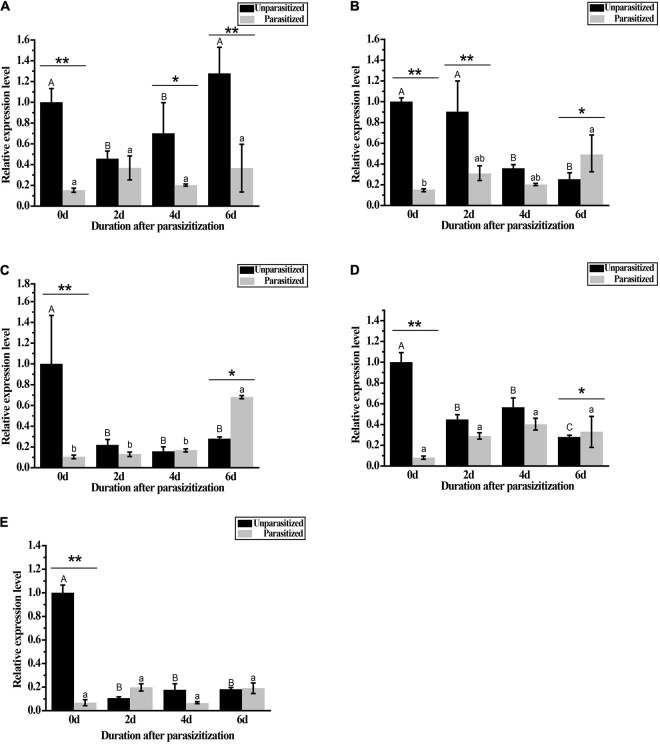
Expressions of *GpHsps* after parasitization by *A. confusum*. **(A)** *GpHsp71.3*; **(B)** *GpHsp74.9*; **(C)** *GpHsp82.4*; **(D)** *GpHsp89*; **(E)** *GpHsp93.4*. The black bar represents the healthy groups and the gray bar represents the parasitization groups. Data are shown as the mean ± SE of three biological replicates. Different upper and lower case letters indicate significant differences between healthy and parasitization groups within the different days (*p* < 0.05). Single asterisks (*p* < 0.05) and double asterisks (*p* < 0.01) indicate significant differences between the healthy and parasitization groups within the same day.

The results showed that the parasitization by *A. confusum* and significantly regulated the expression levels of all candidate *Hsps* in *G. pyloalis* larvae and this moderation were affected by durations post-parasitization (The Two-way ANOVA: for *GpHsp71.3*, *F*_parasitization_ = 68.82, *p* < 0.001, *F*_time_ = 6.82, *p* < 0.01, *F_parasitization_*_×_ *_time_* = 7.15, *p* < 0.01; for *GpHsp74.9, F_parasitization_* = 52.62, *p* < 0.001, *F*_time_ = 11.60, *p* < 0.001, *F_parasitization__×__time_* = 29.45, *p* < 0.001; for *GpHsp82.4, F_parasitization_* = 8.09, *p* < 0.01, *F*_time_ = 9.81, *p* < 0.001, *F_parasitization__×__time_* = 25.82, *p* < 0.001; for *GpHsp89, F_parasitization_* = 31.56, *p* < 0.001, *F*_time_ = 7.49, *p* < 0.01, *F_parasitization__×__time_* = 22.72, *p* < 0.001; for *GpHsp93.4, F_parasitization_* = 83.81, *p* < 0.001, *F*_time_ = 55.43, *p* < 0.001, *F_parasitization__×__time_* = 83.28, *p* < 0.001). All *GpHsps* expressed significantly lower on the first day after parasitization than on other time points. The expression level of *GpHsp71.3* significantly decreased compared to the control group except for the second day after parasitization. The expression levels of *GpHsp74.9 GpHsp82.4*, and *GpHsp89* increased significantly at sixth day after parasitism (Figure 8).

### The Analysis of the Function of *GpHsps* in the Thermal Stress Using RNAi

**FIGURE 9:**
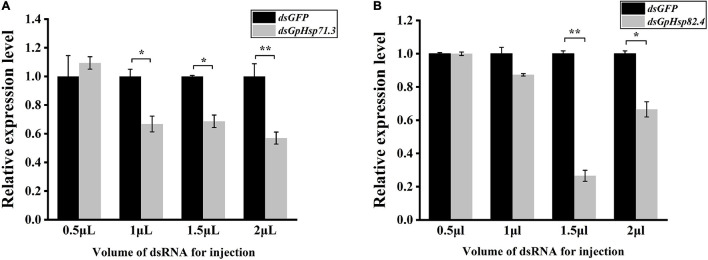
The analysis of the expression of two selected *GpHsp* genes after the knockdown at different injection volumes. **(A)** *GpHsp71.3*; **(B)** *GpHsp82.4*. Data are shown as the mean ± SE of three biological replicates. Differences in the expression levels of each target were compared using a one-way analysis of variance (Systat, Inc., Evanston, IL, United States) with Tukey’s *post hoc* test using R version 4.0.4. Significant differences are indicated by asterisks (*p* < 0.05).

To further investigate the function of *GpHsps* in the thermal tolerance, *GpHsp71.3* and *GpHsp82.4* were selected for RNAi. The results showed that expression levels of both *GpHsp71.3* and *GpHsp82.4* were significantly decreased at 1.5–2 μL injection volume. In particular, the *GpHsp71.3* mRNA level was significantly reduced at 2 μL injection volume compared to the control group. The *GpHsp82.4* mRNA level was significantly downregulated at 1.5 μL injection volume compared to the control group (Figure 9).

### Mortality of RNAi-Treated *G. pyloalis* Larvae to High Temperature Stress

**FIGURE 10:**
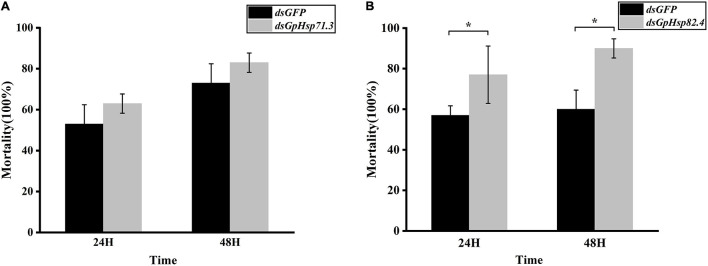
Mortality of *G. pyloalis* larvae to temperature stress after RNAi for 24/48 h. **(A)** *GpHsp71.3*; **(B)** *GpHsp82.4*. Reduction in heat tolerance of *G. pyloalis* injected with dsRNA and then exposed to 40°C (high temperature stress). Data are shown as the mean ± SE of three biological replicates and each replicate consists of ten individuals. Single asterisks (*p* < 0.05) indicate significant differences between dsGFP and dsGpHsp group.

In heat shock stress bioassay, we injected 2 μL *dsHsp71.3* and 1.5 μL *dsHsp82.4* into the individual larva, respectively, according to the optimal injection volume mentioned above. Compared to *dsGFP* control, knockdown of *GpHsp71.3* and *GpHsp82.4* significantly decreased the survivability of *G. pyloalis* larvae to high temperature stress for both 24 and 48 h (Figure 10). The knockdown of *GpHsp71.3* and *GpHsp82.4* resulted in greater mortalities after heat stress, suggesting that these genes are associated with improved protection from high temperature stress in *G. pyloalis* larvae.

## Discussion

Five Hsp genes from the *G. pyloalis* transcriptome dataset were identified, including two Hsp70 family genes (*GpHsp71.3* and *GpHsp74.9*) and three Hsp90 family genes (*GpHsp82.4*, *GpHsp89*, and *GpHsp93.4*). The deduced protein, *GpHsp71.3*, and *GpHsp74.9* have extremely high homology with *Pyrausta nubilalis* and *S. exigua* Hsp70 genes, indicating that insect Hsp70 has a high degree of interspecies conservation (Choi et al., 2014). Furthermore, the C-terminal end of these Hsp70s contains the highly conserved cytoplasm-specific EEVD motif, indicating that *GpHsp70s* functions in the cytoplasm (Gunter and Degnan, 2007; Zhang and Denlinger, 2010). This result is consistent with *Galeruca daurica*, *Anaphothrips obscurus*, *Nilaparvata lugens*, and *Agrotis cnigrum* in the previous studies (Wang et al., 2015; Lu et al., 2017; Tan et al., 2017; Guo and Feng, 2018).

Studies have found that Hsp play a crucial role in regulating insect growth, development, and metamorphosis (Gunter and Degnan, 2007). The expression level of *Hsps* can be expressed at various developmental stages. For example, the expression level of *LsHsp70* in *Laodelphax striatellus* is higher in the first instar larvae (Zhang et al., 2014). The expression level of *GdHsp70* is higher in eggs and first instar larvae of *G. daurica* (Tan et al., 2017). In *Dastarcus helophoroides*, *DhHsp69.09* has the highest expression in the first instar larvae, and *DhHsp70.11* and *DhHsp71.88* are expressed highest in male adults (Zhang Z. et al., 2016). In the present study, *GpHsp71.3* and *GpHsp74.9* were highly expressed at diapause stage. The larvae of *G. pyloalis* will construct pupal chambers to molt into the diapause stage to successfully overwinter (Chen et al., 2015). The high expression of *GpHsp71.3* and *GpHsp74.9* in diapausing larvae indicates that the *Hsp70* may play an important role in the successful overwintering process of *G. pyloalis*. Besides, the expression level of *GpHsp71.3* in pupal stage was higher, and the expression level was significantly increased compared with that in pre-pupal stage, suggesting that *GpHsp71.3* may play a crucial role in the metamorphosis process. This result is similar to Zhang B. et al. (2016) in *Grapholitha molesta*. In *G. molesta*, *GmHsp70* can be highly expressed in diapausing larvae and can also be significantly expressed during the pupal stage. Similarly, the expression levels of Hsp90 genes in different insects are inconsistent with the changes in different developmental stages of insects. In *L. striatellus*, *LsHsp90* has the high expression level in the third fourth instar larvae, and male adults (Zhang et al., 2014). In this study, the three *GpHsp90* genes were highly expressed at prepupal stage followed by pupal stage. A similar scenario is also found in the *Sitophilus zeamais*. The *SzHsp90* is expressed highest at the pupal stage (Tungjitwitayakul et al., 2015).

In many insects, the expression of the Hsp70 and Hsp90 genes is altered in response to temperature stress. The current study shows that heat/cold stress tends to induce high expression of Hsp70 and Hsp90 in insects. In *C. pomonella*, the expression of *CpHsp70-1*, *CpHsp70-2*, and *C. pomHsp90* were all significantly increased in the heat-stressed state (40°C) (Yin et al., 2006). *P. xylostella* showed a significant increase in the expression of *PxHsp70* and *PxHsp90* after heat stress (37°C) (Zhang et al., 2015), and *R. prolixus* showed a significant rapid increase in the expression of *R.proHsp70* under both cold stress (0°C) and heat stress (40°C) (Paim et al., 2016). The trend in the expression of *NlHsp70* at different temperatures showed a continuous increase from 0 to 26°C, while the expression of *NlHsp70* did not change at temperatures from 26 to 40°C (Lu et al., 2017). To evaluate the effect of exposure time on gene expression, two times of the exposure (1 and 4 h) were examined for their short- and long-term effects. The expression of all five *Hsps* in this study was elevated under heat stress, with *GpHsp71.3* and *GpHsp89* being significantly upregulated after 4 h of heat stress. *GpHsp74.9*, *GpHsp82.4*, and *GpHsp93.4* were all upregulated after 1 h of heat stress. The divergence of expression levels of *Hsps* which depending on exposure time is also reported in other insects. For example, in *Helicoverpa zea*, *Hsp90* is expressed at a higher level after a 30 min heat shock, while *Hsp70* reaches its expression peak within 15 min heat exposure, which indicating that *Hsp70* is more sensitive than *Hsp90* to thermal stress in *H. zea* (Zhang and Denlinger, 2010). After 4 h of low-temperature stress, the expression of both *GpHsp74.9* and *GpHsp89* increased, and prolonged low-temperature induced the expression of *GpHsp74.9* and *GpHsp89* genes, presumably playing an essential role in the resistance of *G. pyloalis* to cold stress. Detail mechanism of the effects of long term cold stress on the expression levels of *Hsps* needs to be elucidated in the future study.

When faced with starvation stress, insects change their behavior and physiology to regulate the maintenance of a low metabolic state. The Hsp70 family plays role in response to starvation stress in insects. For example, In *Harmonia axyridis, HaraxHSP68, HaraxHSP70A*, and *HaraxHSP70B* reached maximal levels after starvation (Shen et al., 2015). In *P. puparum* expression of two Hsp genes *PpHsp90* increased after 24 h starvation (Wang et al., 2012). In the starvation-treated *Aphaenogaster picea*, the expression level of *ApHsp40* was twofold change higher than that of the control (Nguyen et al., 2017). We investigated the changes in the expression of *GpHsp70s* and *GpHsp90s* of *G. pyloalis* at different times after starvation and found that *GpHsp74.9* expression was downregulated after 2 and 4 days of starvation. It has been shown that short-term starvation can affect protein homeostasis and nutrient balance in an organism’s cells, leading to a reduction in protein synthesis and nutrient content in the organism (Han et al., 2012). The synthesis and activity of Hsp70 require high energy consumption (Jahangirizadeh et al., 2018). We observed that in the absence of nutrients, the synthesis capacity of *GpHsp74.9* was reduced, leading to a decrease in the expression of *GpHsp74.9*. The expression of *GpHsp82.4* and *GpHsp93.4* followed the same trend, with no significant in expression at the beginning of starvation (2 and 4 days) and a significant increase in expression after 6 days of starvation. It can be speculated that Hsp90 acts as a stress-protective protein and high expression of these proteins are effective in protecting *G. pyloalis* during starvation.

The *G. pyloalis* encounters many natural enemies in its natural environment. Clarifying the molecular response mechanism of the *G. pyloalis* after being parasitized by parasitic wasps can provide an effective, green, and safe strategy and a new method for the biological control of *G. pyloalis*. In this study, the *G. pyloalis* was parasitized by a parasitic wasp, *A. confusum*, and its Hsp70 family and Hsp90 family genes expression levels were measured. The result showed all *Hsps* of *G. pyloalis* downgraded significantly on the first day after parasitization, indicating that the parasitic behavior by *A. confusum* had a significant inhibitory effect on the expression of *Hsp70* and *Hsp90* families in *G. pyloalis*. Among them, the expression level of *GpHsp71.3* was also significantly lower than that of the control after 4 and 6 days of parasitism, and the expression of *GpHsp74.9* was also significantly lower than that of the control group after 2 days of parasitism. Compared to unparasitized larvae of first day, the expression of *GpHsp74.9*, *GpHsp82.4*, and *GpHsp89* was significantly higher in the parasitized group after 6 days of parasitization. Our results showed that the expression level of the six *GpHsps* were significantly influenced by parasitism, and these effects depended on the developmental duration after parasitization. This result is similar to the results found in *S. crassipalpis* (Rinehart et al., 2002). Comparably, after *P. rapae* was parasitized by *P. puparum*, the expression of *PrHsp90* continued to decrease within 48 h, which was significantly different from the control (Zhu et al., 2013). Downregulation of transcripts encoding *Hsp90* in the flesh fly *S. crassipalpis* in response to venom stung by the ectoparasitoid wasp *N. vitripennis* have observed (Rinehart et al., 2002), and this result is similar to the present study. Besides, time duration post-parasitization is needed to be considered since the expression of *GpHsp74.9*, *GpHsp82.4*, and *GpHsp89* was significantly higher after 6 days of parasitization. The possible reason for the downregulation of gene expression in the early stages of parasitism is that the parasitic wasp effectively suppresses the development of the host after injection of parasitic factors while disrupting the host’s immune system, which is largely suppressed by Hsps as an effective immune pathway for the host. Parasitic wasps inject venom and other parasitic factors into the host body while parasitizing (Moreau and Asgari, 2015). These factors cause infection of host blood cells, morphological and ultrastructural changes, apoptosis, inhibition of coagulation and encapsulation reactions, and alteration of actin cytoskeleton structure (Stettler et al., 1998; Carton et al., 2008). To date, parasitic wasp larvae have been shown to use a variety of strategies, such as inhibiting host development, to provide a suitable environment for their growth (Beckage and Gelman, 2004). Studies have shown that there is a functional relationship between hormones and the heat shock regulation system (Lezzi, 1996). Thus, parasitism-induced changes in the expression pattern of the host Hsp70 and Hsp90 genes of *G. pyloalis* suggest that these genes play a vital role in the parasitic process. It is implied that parasitic wasps may use maternal secretions to regulate these genes to create a suitable environment for parasitism. This may be one of the mechanisms by which parasitic wasps manipulate the development of their hosts. Further research is needed on the use of natural enemies such as parasitic wasps in controlling *G. pyloalis.*

## Conclusion

In this study, two Hsp70 genes (*GpHsp71.3* and *GpHsp74.9*) and three Hsp90 genes (*GpHsp82.4*, *GpHsp89*, and *GpHsp93.4*) were identified. We found five GpHsp genes that may be associated with diapause and pupation. Moreover, high-temperature environments can induce the upregulation of gene expression in different degrees, and parasitoid wasps can alter the expression of *Hsp* of *G. pyloalis*. This study presents a new idea for the comprehensive management and biological prevention and control of *G. pyloalis*. In conclusion, *GpHsps* is induced in response to both environmental and biotic stress and plays an important role in the physiological adaptation of *G. pyloalis*.

## Data Availability Statement

The datasets presented in this study can be found in online repositories. The names of the repository/repositories and accession number(s) can be found below: https://www.ncbi.nlm.nih.gov/, SRR9959773.

## Author Contributions

SS and F-aW conceived and designed the experiments. J-hD, L-xZ, JC, and X-hL performed the experiments. J-hD, X-wG, and SS analyzed the data. J-hD, X-wG, and JW contributed reagents, materials, and analysis tools. J-hD and SS wrote the manuscript. All authors have read and approved the submitted manuscript.

## Conflict of Interest

X-wG is employed by Zhenjiang Runyu Biological Science and Technology Development Co., LTD. The remaining authors declare that the research was conducted in the absence of any commercial or financial relationships that could be construed as a potential conflict of interest.

## Publisher’s Note

All claims expressed in this article are solely those of the authors and do not necessarily represent those of their affiliated organizations, or those of the publisher, the editors and the reviewers. Any product that may be evaluated in this article, or claim that may be made by its manufacturer, is not guaranteed or endorsed by the publisher.

## Footnotes

1
https://www.ncbi.nlm.nih.gov/orffinder/

2
https://web.expasy.org/compute_pi/

3
http://pfam.xfam.org/

4
http://smart.embl-heidelberg.de/

5
https://www.ncbi.nlm.nih.gov/tools/primer-blast/

6
https://rnaidesigner.thermofisher.com/
