# Supplementary material for: Characterization, and Functional Analysis of Hsp70 and Hsp90 Gene Families in Glyphodes pyloalis Walker (Lepidoptera: Pyralidae)
Source: Front Physiol. 2021 Oct 21;12:753914. doi: 10.3389/fphys.2021.753914 (PMC8572055; doi:10.3389/fphys.2021.753914)
Supplement: Supplementary file 1 [file Data_Sheet_1.docx]

Supplementary Material

# Supplementary Figures and Tables

## Supplementary Tables

**Table S1.** Primers used for real time RT-qPCR

| **Name** | **Primer sequence（5'–3'）** | **Production size** |
| --- | --- | --- |
| GpHsp71.3F | TCCTTAACGTGTCTGCCGTC | 111 |
| GpHsp71.3R | CTTCGTTAACCATGCGCTCG |  |
| GpHsp74.9F | CTAAGTTGCGCGAGTTGCTC | 128 |
| GpHsp74.9R | TTCAGCCGCCATCTTCTTGT |  |
| GpHsp82.4F | GACGCCCTAGACAAGATCCG | 115 |
| GpHsp82.4R | TGATCGTCAGCGTACCTTCG |  |
| GpHsp89F | GTGGACGAGTACTGCCTGTC | 118 |
| GpHsp89R | ACTCCTCCAGCTGCTCCTTA |  |
| GpHsp93.4F | ACGCGAACAGTAGCCAAGAA | 115 |
| GpHsp93.4R | TGTTGCGGCTCTCCTTTCAT |  |
| GAPDHF | ATTGACAAAGCCTCCGCTCA | 116 |
| GAPDHR | GAGGGGTCATAGGCGTCAAG |  |

**Table S2.** Primers used to synthesize dsRNA.

| **Gene Name** | **Primer sequence（5'–3'）** |
| --- | --- |
| Oligo-1Hsp71.3 | GATCACTAATACGACTCACTATAGGGCCACCGTCCAAGCTGATATTT |
| Oligo-2Hsp71.3 | AAATATCAGCTTGGACGGTGGCCCTATAGTGAGTCGTATTAGTGATC |
| Oligo-3Hsp71.3 | AACCACCGTCCAAGCTGATATCCCTATAGTGAGTCGTATTAGTGATC |
| Oligo-4Hsp71.3 | GATCACTAATACGACTCACTATAGGGATATCAGCTTGGACGGTGGTT |
| Oligo-1Hsp82.4 | GATCACTAATACGACTCACTATAGGGGCGAAAGAACAACATCAAATT |
| Oligo-2Hsp82.4 | AATTTGATGTTGTTCTTTCGCCCCTATAGTGAGTCGTATTAGTGATC |
| Oligo-3Hsp82.4 | AAGCGAAAGAACAACATCAAACCCTATAGTGAGTCGTATTAGTGATC |
| Oligo-4Hsp82.4 | GATCACTAATACGACTCACTATAGGGTTTGATGTTGTTCTTTCGCTT |
| Oligo-1GFP | GATCACTAATACGACTCACTATAGGGGGGATGTCTCACATCTTGTTT |
| Oligo-2GFP | AAACAAGATGTGAGACATCCCCCCTATAGTGAGTCGTATTAGTGATC |
| Oligo-3GFP | AAGGGATGTCTCACATCTTGTCCCTATAGTGAGTCGTATTAGTGATC |
| Oligo-4GFP | GATCACTAATACGACTCACTATAGGGACAAGATGTGAGACATCCCTT |
